# Emergency neurological care of strokes and bleeds

**DOI:** 10.4103/0974-2700.58662

**Published:** 2010

**Authors:** Dale Birenbaum

**Affiliations:** Academic Chairman & Program Director, Florida Hospital Emergency Medicine Residency Program, 7727, Lake Underhill Road, Orlando, Florida 32822, USA

**Keywords:** Cerebrovascular disease, stroke mortality rates, Barthel index, Rankin scale, Glasgow outcome scale, NIHSS, STICH, eloquent brain, ISAT, ISUIA

## Abstract

Ischemic stroke and brain hemorrhage are common and challenging problems faced by emergency physicians. In this article, important details in the diagnosis and clinical management of these neurological emergencies are presented with the following goals: 1) To provide a more comprehensive understanding of the approach to the identification and management of patients who have sustained ischemic and hemorrhagic strokes; 2) to explain the importance and application of commonly used national stroke scoring and outcome scales; 3) to improve the ability to recognize important aspects in the approach and comprehensive treatment of ruptured and unruptured intracranial aneurysms; and 4) to demonstrate the difficulties in the neurological, neurosurgical, and endovascular treatment of these catastrophic diseases.

## INTRODUCTION

This article will focus on the clinical approach to ischemic stroke, intracerebral intraparenchymal hemorrhagic stroke, aneurysmal subarachnoid hemorrhage, ruptured intracranial aneurysms, and unruptured intracranial aneurysms. Important details in the diagnosis and clinical management of these neurological emergencies are presented in order to stimulate further discussion with colleagues in neurology and neurosurgery so that rational treatment plans may be developed. This article attempts to clarify the approach to the clinical management of these neurological emergencies.

Stroke can be a devastating disease. The overall mortality rate is >20%. It is the third leading cause of adult deaths in the United States and is the leading cause of permanent disability.[1–5] National mortality rates for penetrating trauma remain less that 5% and level-1 trauma centers are widely supported and have the resources and infrastructure to have in-house expert trauma teams and operating rooms that are always available.[1–5] While the initial presentation of the stroke patient may not be as dramatic as that of the patient who presents with a stab wound to the chest, this is clearly a disease that should receive similar attention and not be underestimated. In order to reduce morbidity and mortality due to this devastating disease, the development and implementation of designated primary and comprehensive stroke centers has become increasingly important.[2–5] The goal is to focus on stroke care as there have been significant advances in the management of this condition over the last decade.

## ISCHEMIC STROKE

Within the first few hours of the onset of an ischemic stroke, the interventional neuroradiologist can sometimes remove a large occluding clot from the cerebral circulation. Early reperfusion allows the area of the ischemic penumbra to be saved and this could allow for a faster improvement in the clinical outcome. However, this is not always possible. It can take months for patients to make a modest recovery, which only emphasizes the fact that the effects of ischemia, reperfusion, and reperfusion injury are not yet fully understood.

Success in the treatment of ischemic stroke seen with the use of intravenous tissue plasminogen activator (TPA) in the National Institute of Neurological Disorders and Stroke (NINDS) trial was measured at 3 months, not immediately.[2–6] Patients who make a more rapid recovery might have been experiencing a reversible ischemic neurologic disorder (RIND).

## CLINICAL APPROACH TO ACUTE ISCHEMIC STROKE

Ischemic stroke is a devastating disease with the only FDA-approved treatment being intravenous (IV) thrombolytics administered within 3 h from the time the patient was last seen to be normal.[2] The MERCI clot-retrieval system is also an FDA-approved device that can be used by trained interventional neuroradiologists and neurosurgeons to aid stroke treatment; it can be used for up to 8 h after the patient was last seen normal.[2,7]

Unenhanced CT scan is the initial rapid study of choice and can help identify early signs of stroke and to rule out hemorrhage. It is also used to identify signs of a large early infarction and to identify patients with less than one-third middle cerebral artery (MCA) hypodensity. Patients with signs of large early infarcts on the initial CT scan may be at an increased risk of an adverse outcome, while those patients whose CT scan shows hypodensity greater than one-third of the MCA territory have an increased risk of bleeding complications.[2–7] With all ischemic strokes the hemorrhagic risk increases with time, and the sooner patients are treated the better is their chance of a good outcome.

Standardized stroke assessment scales are used throughout the world in order to quantify patients' initial clinical condition and to measure clinical improvements. Below is a description of the four most commonly used scales.

The *National Institute of Health Stroke Scale (NIHSS)* is a 42-point scale, with the score ranging from 0–42. It is based on the evaluation and recording of 11 specific details tested on the neurological examination. A low total composite score indicates a mild stroke, while a high score indicates a severe stroke. The initial NIHSS stroke score also has significant predictive value. Patients with a NIHSS stroke score < 1 appear clinically normal. Those with a NIHSS score > 4 can be considered for treatment.

The *Barthel index* is a 1–100 scoring system that assesses an individual's ability to care for himself/herself, for example, in feeding, toileting, and ambulation. Scores > 95 represent patients who are completely independent.

The *modified Rankin scale* is scored on a scale from 0–5; zero indicates no disability and 5 severe disability.

The *Glasgow outcome scale* is scored on a scale from 1–5, with 1 representing good recovery and 5 representing death.

The original landmark study on the use of tissue plasminogen activator (TPA, specifically alteplase) for ischemic stroke that was published in the *The New England Journal of Medicine* in 1995 by The National Institute of Neurological Disorders and Stroke (NINDS) rt-PA study group[2–7] demonstrated that there was no immediate improvement in patient outcomes. The results of NINDS-part II demonstrated good clinical outcomes at 3 months in 333 patients. There was an absolute increase of 11-13% for a favorable outcome in treated patients. The symptomatic intracerebral hemorrhage rate at 36 hours was 6.4% vs 0.6% in the placebo group. Despite this increased risk of symptomatic intracranial bleeding 3-month mortality rates were similar in the treated (17%) and the placebo (21%) groups.

The magnitude of clinical improvement that was observed in the NINDS trials and recently revalidated in the third European Cooperative Acute Stroke Study, (ECASS-III) and published in the NEJM (September 25^th^ 2008)[2–8] can only be appreciated by having a full understanding of the scoring systems. Patients who met the following outcome scores that are used in the stroke studies mentioned appear clinically normal. The outcomes scores measured as a Barthel index > 95, modified Rankin scale < 1, Glasgow outcome scale = 1, and NIHSS < 1 represent the clinical appearance of patients who have had a dramatic result with treatment despite facing a potentially life-threatening stroke.

The impressive outcomes can be translated into the clinical arena in the following manner: of patients who have sustained an ischemic stroke approximately one-third will stay the same, one-third will progress and die, and one-third will improve.

The use of TPA increases the likelihood of improvement from 30% to 40–50% and, despite the risk of hemorrhage being greater in treated patients, the mortality rates are similar in the treated and the untreated.[2–8] This may occur because perfusion to the area of the ischemic penumbra may be more important than the subsequent reperfusion injury, sometimes manifested as intracranial hemorrhage. It is important to explain this information to patients and their families in order to guide and assist them choose the treatment option.

The recent ECASS-III trial results suggest that the window period for intravenous thrombolytics may be extended to 4.5 h after the onset of stroke symptoms. In all instances, having more time does not mean that we should waste it, because patients will have better outcomes if they are treated earlier. It has also been shown that community hospitals can also safely administer intravenous TPA if strict NINDS protocols are followed.[35]

Stroke experts are now looking for reasons to treat patients with TPA, instead of reasons not to treat. This represents a significant shift in the approach to the treatment of this potentially devastating and life-threatening condition,[2–9] which has often been ignored in the past due to the lack of treatment options.

Angioedema has become a well recognized but rare complication resulting from the administration of TPA.[2] The reaction presents with oral and pharyngeal swelling along with tongue edema, similar to that seen in ACE inhibitor reactions. Prompt recognition of this reaction is essential in order to prevent increased morbidity and mortality. Airway protection, stopping the drug's administration, and treatment with antihistamines and steroids is advised.

The MERCI clot-retrieval system is a mechanical embolectomy device. In the Multi-MERCI trial where the primary endpoint was recanalization of the target blood vessel, the device was effective 57% of the time. Among patients who experienced recanalization, there was a 2-fold survival advantage and a significantly higher proportion of patients lived without significant disability.[10,36]

The trial did not show that thrombectomy actually improves stroke outcome. The 27% reduction in mortality that was observed between recanalizers and nonrecanalizers is evidence that the device works and, by allowing perfusion, seems to improve survival. Many of the patients in the MERCI trials had experienced significantly larger strokes than patients in the original NINDS TPA study and the treatment in some cases was extended up to 8 hours. Further outcome studies are needed to provide more conclusive evidence of the benefits of clot-removing devices.[36]

Additional factors aiding in stroke recovery and survival are listed in Table 1.

**Table 1:** Factors aiding stroke care and survival[2–8]

Factors aiding stroke care and survival
Application of standing order sets
Early mobilization and rehabilitation
DVT prophylaxis
Swallowing assessment prior to feeding
Dedicated stroke units
Rapid response teams

Recommendations for acute blood pressure treatment goals are outlined below. One important target blood pressure number to remember is 185/110, which is the level for entry into acute treatment protocols. Overcorrection and rapid drops in blood pressure can result in a poor outcome. The recommended target blood pressures call for moderate reduction of blood pressure and do not require that normal blood pressure be attained. The initial blood pressure can be lowered to bring the patient in range for the administration of TPA.

Acute blood pressure goals: SBP DBP MAP

Target BP

Not eligible for acute therapies <220 <120 <130 - First 24 h

Pre-treatment thrombolytic/ <185 <110 recanalization therapies

Post-treatment thrombolytic/ <180 <105 recanalization therapies

Decompressive hemicraniectomy, or the process where the skull and one half of the cranium are removed in order to allow for expansion of the residual brain, is a poorly understood life-salvaging technique.[11] It is a technique that should be reserved for patients who have sustained a stroke to their non-dominant hemisphere harboring areas of less eloquent brain. Decompressive hemicraniectomy can saves lives and younger stroke patients do show a greater chance for recovery but unfortunately they are unlikely to become independent.

Modern ischemic stroke care is evolving. Intravenous TPA and the MERCI clot-retrieval system should be considered in all eligible patients. The application of the guidelines listed in Table 1 are helping patients to recover and survive.

Remember, that the sooner a patient is treated the better. Begin to look for reasons to treat with these modalities, instead of reasons not to treat.[2–11] Dynamic CT and MRI scanning, along with telemedicine, are currently being utilized and studied at multiple stroke centers throughout the world. These diagnostic and treatment modalities will have a significant impact on shaping the future of modern stroke care.

## SUPRATENTORIAL INTRACEREBRAL INTRAPARENCHYMAL HEMORRHAGIC STROKE

**Figure 1:**
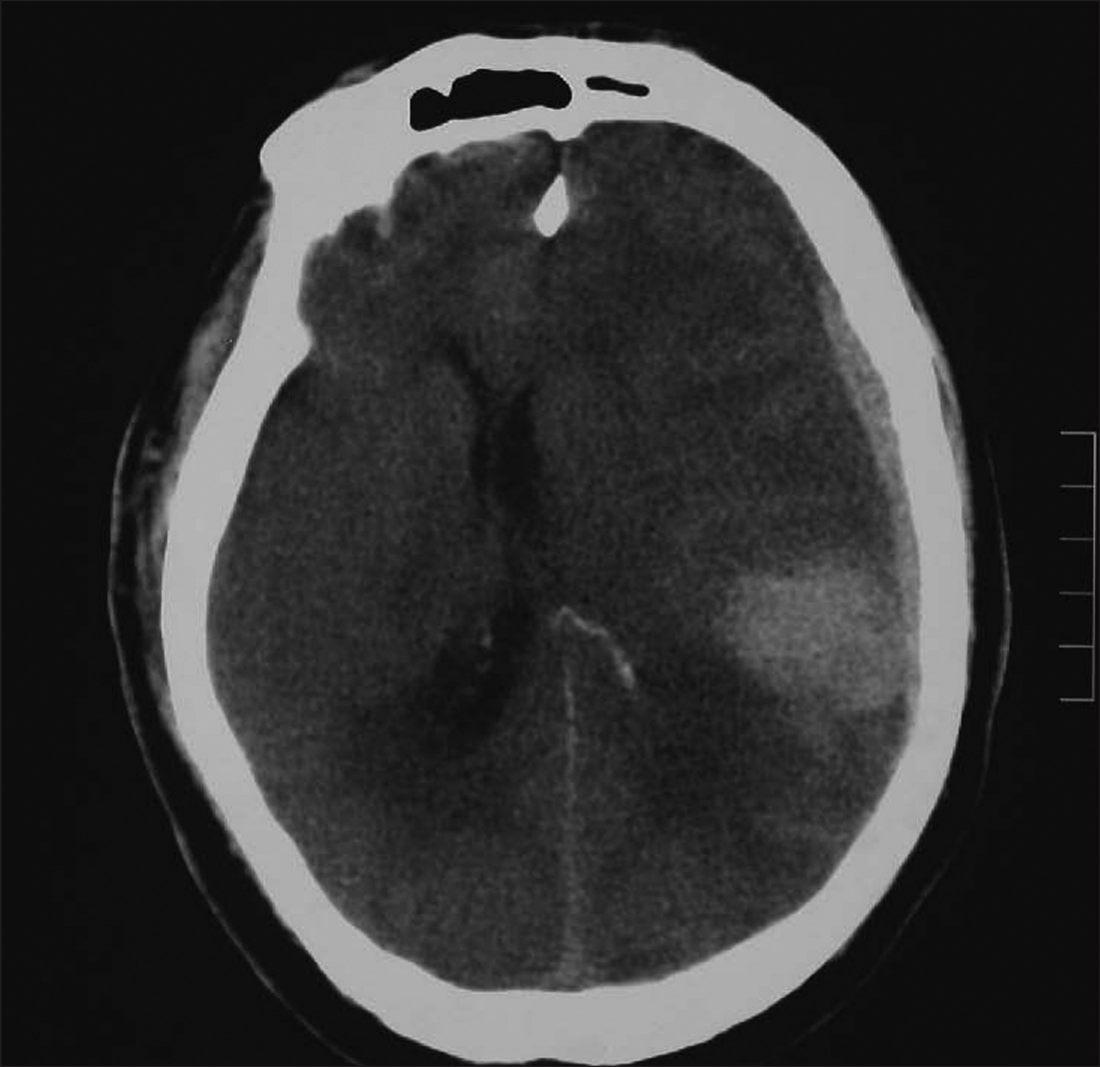
Large intraparenchymal ICH

Supratentorial intracerebral intraparenchymal hemorrhagic strokes are much less common than ischemic strokes, occurring in a frequency of about 20%; however, they are clearly the most lethal form of stroke and account for more than 50% of all stroke deaths[12–14] There is nothing more challenging in emergency medicine than receiving an obtunded patient in whom the CT scan reveals a large intracerebral intraparenchymal hemorrhage as shown Figure 1.

In this case it is important to stabilize the patient by securing the airway, carefully monitor the vital signs, and consider placing an emergency call to the neurosurgeon on call.[12–14] While the etiology of the bleeding is often mixed, it is essential to try and separate out the causes so that one can have a more comprehensive understanding of the management of these conditions in the clinical arena.

Understanding the neurosurgeon's approach to the evaluation and treatment of these patients will improve patient care. The large intracerebral intraparenchymal hemorrhage shown in Figure 1 occurred in the dominant hemisphere of an elderly patient with multiple medical problems and may not be a survivable insult even with optimal medical and surgical treatment.

## CLINICAL APPROACH TO SPONTANEOUS INTRACEREBRAL HEMORRHAGE

Unlike ischemic stroke in which medical complications are the most common cause of death, the key to reducing death and disability caused by spontaneous intracerebral intraparenchymal hemorrhage is to act as rapidly as possible to identify the size and location of the bleeding and to find out the level of function prior to the insult. Understanding the importance of tight blood pressure control and the need to maintain adequate cerebral perfusion pressure, undertaking measures aimed at reducing hemorrhagic enlargement, and being aware of the indications for surgical evacuation will help in the management of the patient.[12–14]

The initial clinical presentation of a patient who has sustained spontaneous intraparenchymal hemorrhage has important prognostic implications. The patient who appears clinically normal has a much better chance of survival than those who present with sudden death or rapidly progesssing neurological deterioration. Patients who are drowsy and stuporous fare better than individuals who present in a moderate to severe coma. Prognosis is also strongly related to the size of the initial hemorrhage. The patient who has an initial hemorrhage volume of more than 85 ml has an extremely high mortality rate that is almost uniformly fatal. An initial bleeding volume of greater than 50 ml is also extremely serious with mortality rates approaching 76 to 90%.

The location of the intracranial bleeding is also significant and can help determine prognosis. When the intracranial bleeding is lobar, large, and extends into the ventricles, mortality rates approach 80–90%. In contained, relatively deep thalamic and putamen intracerebral intraparenchymal hemorrhage, the mortality rates are significantly lower at 20–40%. Subcortical lobar intracerebral hemorrhagic strokes that are located within 1–2 cm of the brain's surface and are less than 2–3 cm in size present in patients with milder deficits and have the best prognosis, the mortality rate being only 10%.

After the initial assessment and evaluation is made, efforts are focused on trying to reduce hemorrhagic enlargement. Adequate blood pressure control is important in reducing hemorrhagic enlargement. Hemorrhagic enlargement occurs in approximately 30–40% of intracerebral hemorrhagic stroke patients and increases the chance of a poor outcome from 50% to 80%. The goal in managing the blood pressure is to avoid large fluctuations and dramatic shifts. This can be accomplished by having an intimate awareness of the blood pressure and of any agents that could have an effect on it.

The goal in effectively managing the blood pressure is to have adequate blood flow to potential areas of the ischemic penumbra; therefore, it is essential to maintain a cerebral perfusion pressure (CPP) >60 mm/Hg The mean arterial pressure (MAP) minus the intracranial pressure (ICP) is equal to the cerebral perfusion pressure (CPP). MAP = diastolic blood pressure plus one-third the difference of the systolic and diastolic blood pressure (SBP–DBP). For example, in a patient with a BP of 120/80, the MAP = 80 + 13.3 [i.e., ⅓ (120–80)] = 93.3. Calculation of the CPP requires measurement of the ICP. The blood pressure can be modified if it is high or low.[12–14]

If the initial blood pressure is extremely high (>250/130), it will be essential to use an intravenous agent like nicardipine that can be easily titrated and is reliable. The initial goal should be a reduction of the blood pressure by no more than 15%, as many of these patients would have accommodated to higher baseline blood pressures and rapid reductions may lead to worsening of the ischemia.

Patients who present in shock may require an initial fluid bolus followed by an intravenous pressor such as norepinephrine to elevate a low arterial pressure of < 70–80 mm Hg to about 130 or 140 mmHg, so as to allow for enough of a gradient between mean arterial pressure and intracranial pressure to maintain adequate cerebral perfusion. An enhanced external counterpulsation (ECP) device might also be considered to elevate arterial pressure if the patient presents in hypotension.[12–14] ECP is an approved noninvasive therapy for angina, congestive heart failure, myocardial infarction, and cardiogenic shock that augments blood flow to cardiac and systemic circuits. ECP is a physical device used to augment blood pressure. When timed to the patients heart rate air-filled cuffs are inflated around the lower legs during diastole The hemodynamic effects of ECP include 20–25% improvement in blood volume in the carotid, renal, and hepatic arteries. ECP's hemodynamic effects are similar to that obtained with intraaortic balloon pumps (IABP), but carry less implementation risk as the procedure is noninvasive.

There was initial hope that recombinant factor VIIa (RFVIIa) would also help reduce hemorrhagic enlargement as its use showed reduction in hematoma growth and mortality along with some improved global outcome scales at 90 days.[15,16] The recombinant activated factor VIIa phase-III FAST (rFVIIa in Acute Hemorrhagic Stroke Treatment) international trial ended early because of increased mortality in the treated group. The complications and increased mortality rates seen with RFVIIa are thought to be related to arterial thromboembolic events.[15,16] These events include ischemic stroke, myocardial infarction, and vascular insufficiency and emphasize the fine balance between bleeding, perfusion, and ischemia.

For patients who sustain spontaneous intracerebral intraparenchmal hemorrhage and are on anticoagulants such as warfarin (Coumadin) and heparin, it is important to aggressively reverse the effects of these anticoagulants in order to prevent hemorrhagic enlargement.[17] For patients who are receiving warfarin it will be necessary to infuse fresh frozen plasma (FFP) or prothrombin complex concentrates (PCCs) as soon as possible once the diagnosis is established and to consider repeating if necessary prior to transfer. Patients on heparin can be reversed with protamine sulfate.

## SURGICAL EVACUATION AND CRANIOTOMY FOR INTRAPARENCHYMAL INTRACEREBRAL HEMORRHAGE

Craniotomy is typically performed for signs of increased intracranial pressure as manifested by a mass effect or midline shift and the absence of basal cisterns on the CT scan; it is done only after taking into account the age and level of function prior to the event.[13,14] Young patients and those functioning at higher levels generally tend to do better. There is absence of general agreement on the best time and indications for surgery. One thing that is clear in the operative management of intracerebral hemorrhage (ICH) is that hydrocephalus caused by intraventricular hemorrhage is treated with CSF drainage and ventriculostomy, sometimes aided by intrathecal lytics.[18] The international CLEAR trial is an ongoing multicenter study evaluating the utility of intraventricular thrombolytics and is showing evidence of improvement in hemorrhagic stroke patients.[19] Small doses of thrombolytics are injected directly into the ventricles and help drain clotted blood in the ventricles through a ventriculostomy, thus aiding in the reduction of hydrocephalus and intracranial pressure.

A recent landmark study aids in understanding the use of surgical evacuation for spontaneous ICH.[18] In this study The Surgical Trial of Intracerebral Hemotoma or STICH, Dr David Mendelow of the United Kingdom looked at early surgery *vs* initial conservative treatment in patients with spontaneous, nontraumatic supratentorial ICH who underwent surgery within 24 h, with a median time to surgery of 20 h. Over a 10-year period, 1000 patients were randomized and evaluated.[18] The conclusions revealed that in patients with spontaneous supratentorial ICH the outcome was the same, regardless of whether they had received initial conservative medical treatment or early surgical intervention. STICH researchers also developed an outcome prediction tool that may help guide decisions for therapy in the clinical arena. The results were presented at the International Stroke Association meeting that took place in Orlando, Florida, in 2006.[20] Below is a description of the clinical tool and some examples of its potential for application in the clinical arena.

## DECISION TOOL DERIVED FROM THE CLINICAL TRIAL

Patients are given an initial starting score for ICH of 300; To this add 7 times the patients age, add 90× midline shift in cm, add volume in cc, add 70 if hydrocephalus present, add 40 if intraventricular hemorrhage present, add 90 if worst limb is weak, add 180 if worst limb is paralyzed, add 90 if basal ganglia (bg) or thalmic (thal) bleeding is present, add 120 if both lobar and bg/thal, subtract 20× GCS.

The result of this calculation produces the ‘results score.’ When the results score was > 900, the model predicts that patients had 0% chance of a good to moderate recovery. For results scores < 500 the model predicts that patients will have a 65% chance of a good to moderate recovery.

**Figure 2:**
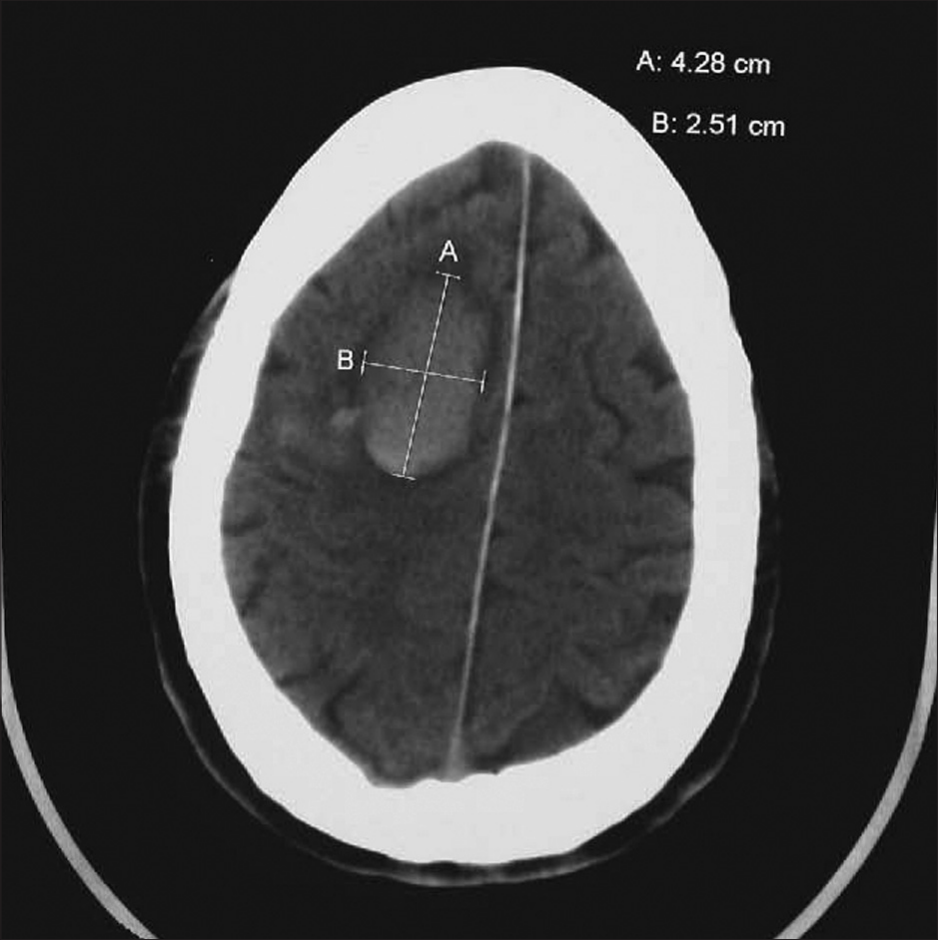
Moderate subcortical intracerebral hemorrhagic stroke

The best predictors in this model for outcome were the neurological deficit, GCS, age, and CT data revealing midline shift, and the site and presence of hydrocephalus.[18,20] This tool can help guide treatment and be of use when decisions are made in consultaion with neurosurgical specialists. The examples presented in Figures 1 and 2 show the results score calculation following this model and its potential application in the clinical arena.

Figure 1 is a large left-sided spontaneous intracerebral intraparenchymal hemorrhage in a 70-year-old man who is not on anticoagulants. The patient presented obtunded, with a large bleed in the dominant hemisphere, with midline shift; he was paralyzed and deeply comatose.

Starting initial ICH score 300. Add 7 times age of 70 (490), 90 times 2-cm shift (180), volume 100 ml (100), limb paralyzed (180), subtract 20 × GCS (60) = Total score of 1190.

The total results score > 1190 obtained by applying the model indicates that this patient has a poor prognosis and that this bleeding will be almost certainly fatal. In this obtunded patient with a large midline shift, the lesion is operatively approachable but is near eloquent brain. The information helped guide the neurosurgeon and family to choose initial conservative management followed by admission and observation. The patient eventually died.

While the model may assist us in making clinical decisions, the need for close observation for the first 24 h in the management of all these patients is stressed in recent guidelines, as some patients can make dramatic and unexpected improvements.[13,14,18–20]

Figure 2 demonstrates a moderate-sized right-sided intracerebral hemorrhagic stroke that occurred in a conscious 40 year- old male patient who appeared a little confused after awakening.

The results score was calculated: Starting initial ICH score 300. Add 7 times age of 40 (280), no midline shift, volume 10 ml (10), no intraventricular hemorrhage, no weakness, no bg/thal, Subtract 20 × GCS (14) (i.e., –280). Results score = 310.

The results score is much less than 500 and the model predicts that the patient has a good prognosis, with 65% chance of good recovery. This was a bleed in the easily accessible frontal area of the brain in a patient who was awake. It was evacuated and treated without difficulty by the neurosurgeon. The prediction tool is not used to score infratentorial hemorrhages as described below.

## INFRATENTORIAL INTRACEREBRAL INTRAPARENCHYMAL HEMORRHAGE

**Figure 3:**
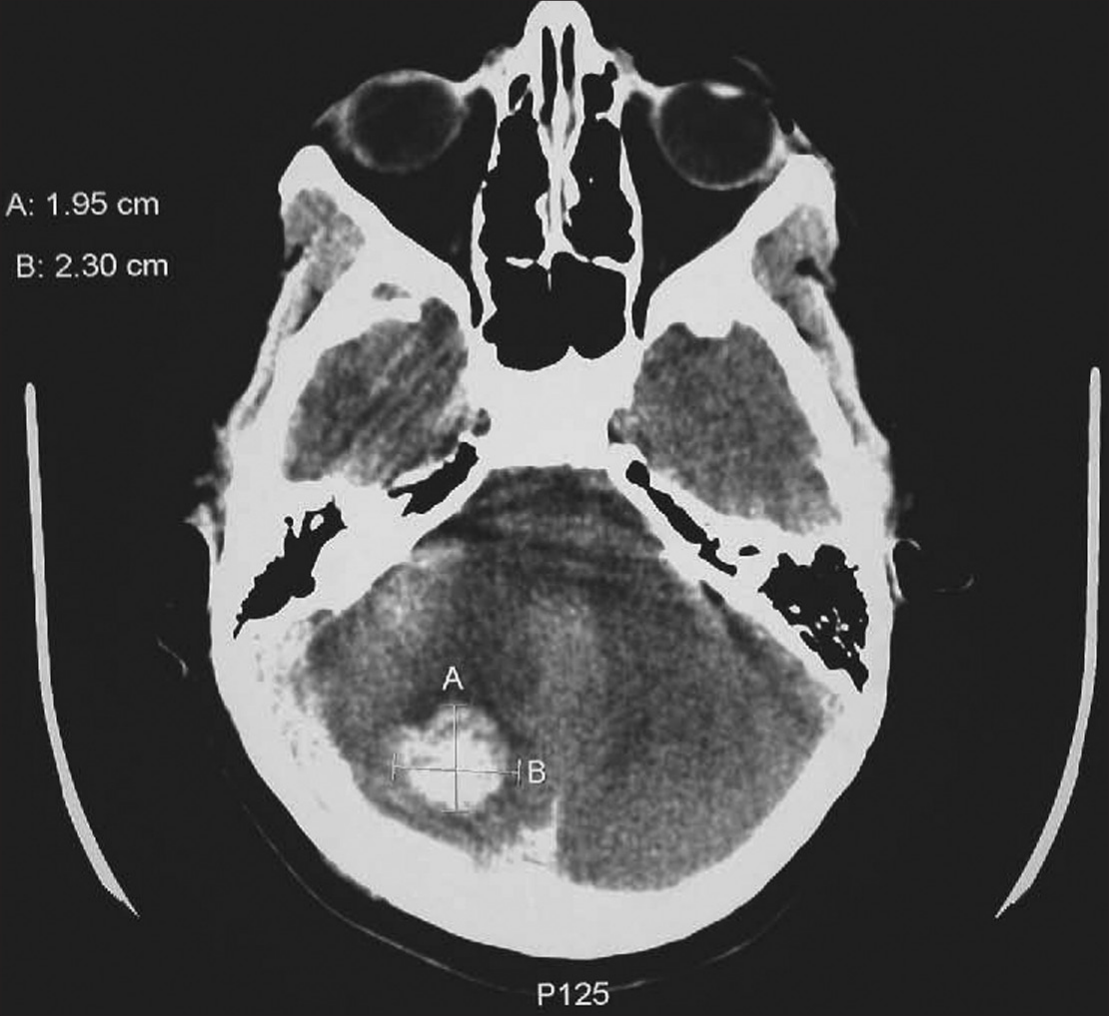
Cerebellar/infratentorial bleed in a child. The only chance for survival is rapid decompression to avoid pressure on the brainstem. The prognosis will be related to rapid access to the operating room and an experienced neurosurgeon

The approach to the management of patients with infratentorial cerebral hemorrhages has some unique features since the space available is smaller. The CT scan in Figure 3 is of a 10-year-old boy with a large right cerebellar hemorrhage. When the bleeding is located in the cerebellum and has a size greater than 3 cm, or if there is a cerebellar hemorrhage with brainstem compression or hydrocephalus, mortality rates may approach 80–90%. Emergent evacuation of the hemorrhage offers the only chance for survival, and clinical outcome will depend on prompt access to a qualified and experienced neurosurgeon, which might not always be readily available. In these patients medical management alone results in bad outcomes.

Smaller cerebellar hemorrhages without brainstem compression can be managed medically and do reasonably well.

## INTRACEREBRAL INTRAPARENCHYMAL HEMORRHAGE PEARLS

Try to maintain adequate cerebral perfusion and avoid large blood pressure fluctuations while treating patients with intracerebral intraparenchymal hemorrhages. Promptly reverse coagulopathies; this will aid in the reduction of hemorrhagic enlargement. Know the general guidelines for surgery as this will facilitate communication with your colleagues in neurosurgery. In all cases, consultation with an experienced neurosurgeon is essential.

General guidelines that indicate the need for early surgery in intracerebral hemorrhage are as follows:[13,14,18,20]

1. Spontaneous supratentorial intracerebral hemorrhage with intraventricular extension and hydrocephalus
2. Contained subcortical hemorrhage
3. Cerebellar hemorrhages greater than 3 cm
4. Cerebellar hemorrhage with brainstem compression or hydrocephalus

## ANEURYSMAL SUBARACHNOID HEMORRHAGE

**Figure 4:**
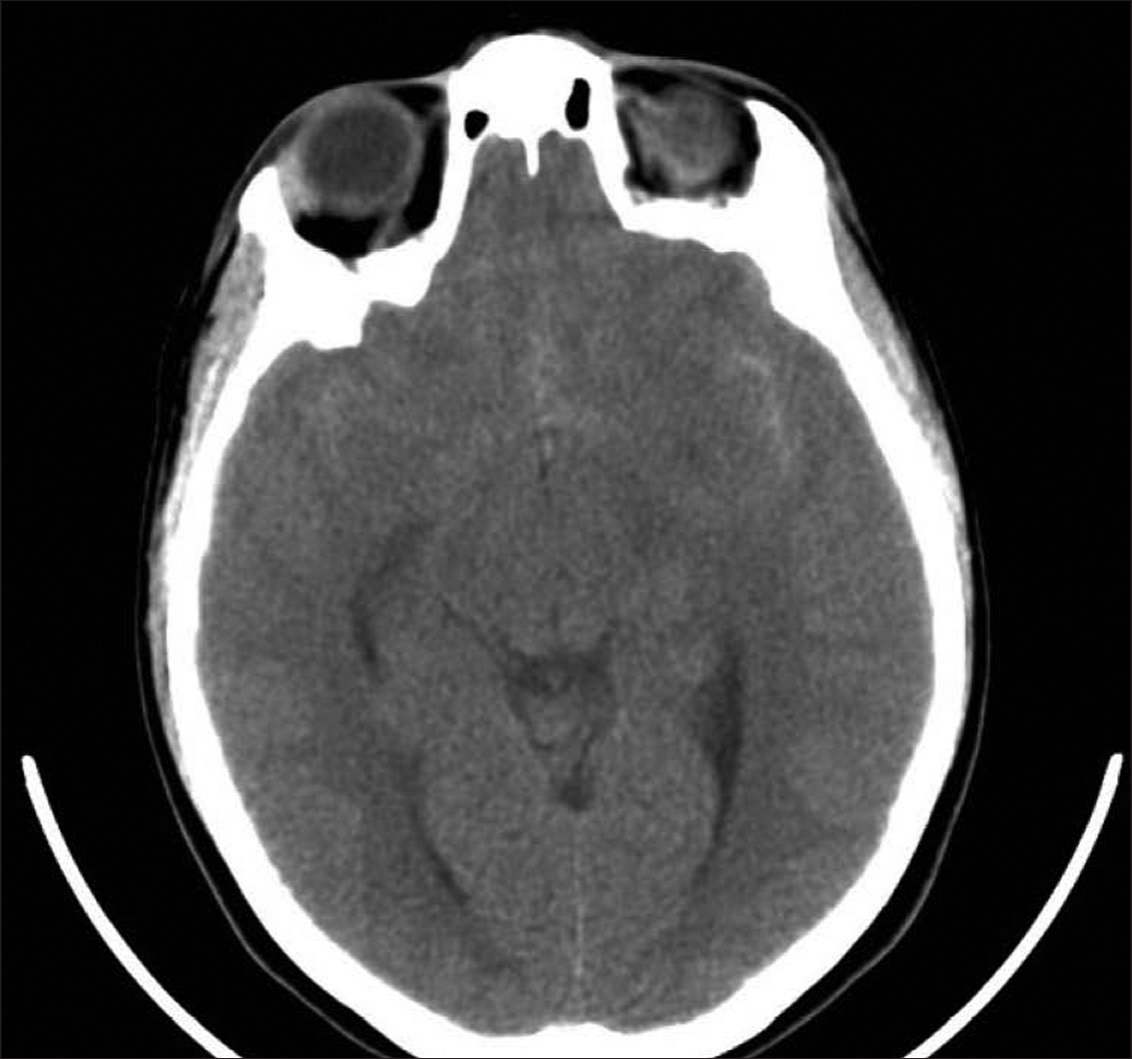
Subarachnoid hemorrhage

Stroke due to aneurysmal subarachnoid hemorrhage (aSAH) is different from other forms of hemorrhagic stroke and remains a daunting challenge to diagnose and treat. This devastating condition accounts for approximately 5% of all strokes but inflicts death or dependence on two thirds of its patients[13,21–23] Example of CT with SAH [Figure 4].

Although trauma is the leading cause of subarachnoid hemorrhage (SAH), ruptured intracranial aneurysms account for 80% of non-traumatic cases. Of the remaining 20%, half are caused by non-aneurysmal venous hemorrhages and the rest by arteriovenous malformations, other vascular lesions, tumors, and rare disorders. The management of aSAH is similar to that of spontaneous intraparenchymal intracerebral hemorrhage, but there are some unique features.

There may be occasions when the bleeding may have a mixed etiology and, in such cases, identification of the different causes allows for a more comprehensive understanding and better management of the case. Initially, it is important to act as rapidly as possible to stabilize the patient and to identify the size and location of the bleeding, along with level of function prior to the insult.

The importance of tight blood pressure control and the need to maintain adequate cerebral perfusion pressure must be understood. Efforts aimed at reducing hemorrhagic enlargement and the indications for surgical evacuation are similar to those for spontaneous intracerebral intraparenchymal hemorrhage. The most important aspect to the management of patients who have sustained an aSAH is the prevention of re-rupture.[13,21–25]

As with other forms of intracranial bleeding, mortality and outcome rates are closely related to the initial clinical presentation. The Hunt and Hess classification (with grades of 1–5) is used to grade aSAH patients.

## HUNT AND HESS CLASSIFICATION

Grade 1: Asymptomatic or minimal headache

Grade 2: Moderate to severe headache, nuchal rigidity, no neuro deficits, except cranial nerves

Grade 3: Drowsiness, confusion, minimal focal neurological deficits

Grade 4: Stuporous; moderate to severe hemiparesis, possible posturing

Grade 5: Posturing, deep coma, moribound

Patients who have a high grade of 4 or 5 at presentation do not generally have good clinical outcomes. The principles of management are the same as in intraparenchymal hemorrhage and focus on optimizing blood flow and balancing bleeding, ischemia, and perfusion. The initial goal is to optimize blood pressure and maintain a cerebral perfusion pressure of 60 mmHg. Rapid shifts and fluctuations are to be avoided and tight blood pressure control helps to prevent hemorrhagic enlargement. Hydrocephalus caused by intraventricular hemorrhage is treated with CSF ventriculostomy drainage. Prompt consultation with a neurosurgeon is essential. Mortality rates in aSAH approach 20–50%, with the highest risk of death and disability being in patients who have rebleeding and re-rupture. Preventing re-rupture is the most important aspect in the management of patients who have sustained an aSAH. Understanding this concept is critical to the success in managing these patients. It is also the reason why we perform lumbar puncture in patients who say they have the worst headache of their lives; the idea being to identify individuals with a small initial or herald bleeding. This investigation has not yet been replaced by MR (magnetic resonance) study.

From the natural history of bleeding aneurysms it appears that the risk of re-rupture is as follows:[21–25]

Within 2 weeks 15–25%

Within 6 months 50 %

Cumulative annual risk after 6 months is an additional 2-3 % per year.

In order to prevent rebleeding a variety of techniques are used and include: surgical correction, embolization, coil, and stent–coil combinations.[26–29] Prior to 1992, when the Guglielmi detachable platinum coil (GDC) was introduced in Europe, there was no alternative to craniotomy and neurosurgical clipping to secure the aneurysm and prevent rebleeding.[37]

Endovascular coiling is the process by which a microcatheter is threaded through a guide catheter up to the origin of the ruptured aneurysm. Once inside the aneurysm, the platinum coils are gently inserted into the sac in a sequential outside-in multilayered fashion until the aneurysm is densely packed. This process relies on a critical volume of embolization and reduced blood flow through the treatment zone.[22]

Patients with coil therapy require serial monitoring and follow-up imaging for prompt detection of the occasional risk of coil compaction or recanalization, which can occur in larger necks or in wide-sized necks and poorly packed aneurysms. With initial treatment, in up to 70% of patients it is possible to achieve 95–100% occlusion of the aneurysm. However, 24–30% of patients do not have complete obliteration of the aneurysm and recanalization can occur, with the rate of subsequent complications being around 10%.[30]

There is a tremendous amount of ongoing neurological and neurosurgical research on the best approach to the treatment of aneurysms. One of the most well recognized studies in this field is the International Symptomatic Subarachnoid Aneurysmal Trial (ISAT). In this study, investigators randomized 2,143 patients with ruptured aneurysms to receive neurosurgical clipping *vs* endovascular coiling. The results were reported in the Lancet in 2002 and found more recovery at 1 year in the endovascular coiling group, as evidenced by an absolute risk reduction of 6.9% as measured by the modified Rankin scale.[26–29] It is important to note that 97.3% of the treated patients had aneurysms in the anterior circulation, with 88% being of the lower grade; also, the majority of the aneurysms were relatively small, with 94% being < 1 cm. The patients who had surgery for ruptured basilar tip (those in the back of the brain) had an 83-84% success rate and for these patients surgery was as good as coiling.

This study does emphasize that the size and location of the aneurysm are critically important and are major factors in determining the chance of recovery and the morbidity and mortality. Individuals who perform endovascular coiling strongly support the results of ISAT, and feel that this noninvasive, non-surgical approach is better and does less damage to adjacent, eloquent brain. While this technique is favored for smaller aneurysms, patients with larger aneurysms may have the risk of recanalization.

Columbia University's neurosurgical team emphasizes that the annual recurrence rate following surgical clipping of an aneurysm is only 0.26–0.52%.[30] Therefore surgical clipping may be a better definitive treatment plan; however, the problem is that not all aneurysms can be reached by open surgical techniques.

The prognosis following microsurgical clipping of aneurysms is also related to the experience of the neurosurgeon. Neurosurgeons who clip less than 30 aneurysms per year have higher operative mortality rates. Therefore some neurologists and neurosurgeons strongly advocate the concept of aneurysm centers.[30] There are already a few in the United States. These specialized high-volume centers have hands-free microscopes with intraoperative video angiography-endoscopy, fenestrated clamps, and years of experience, along with large teams of neurosurgeons who specialize in the care of patients with intracranial aneurysms.

The blood from SAH is irritating to the brain and arterial vasospasm and occlusion can occur, leading to ischemia in the non-injured penumbra.[31] This effect may be at a distance from the artery of aneurysmal rupture and therefore global transcranial Doppler study is indicated to evaluate for vasospasm and is typically performed at day 3 after the initial presentation. If vasospasm is found nimodipine at a dose of 60 mg every 6 h for 21 days is indicated. Nimodipine works by blocking calcium entry into cells and is more of a neuroprotective agent in this clinical setting than it is a vasodilator.

**Figure 5:**
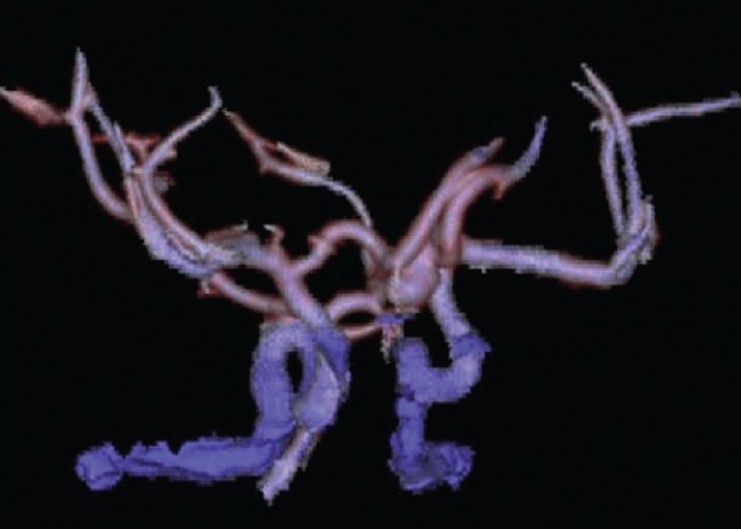
Large ACOM aneurysm

Figure 5 shows a large anterior communicating artery aneurysm visualized on the CT angiogram of a healthy 13-year-old student presenting with a mild headache; the initial CT scan had revealed a small area of SAH. The Hunt and Hess grade was 1. The treating pediatric neurosurgeons were able to stabilize and refer this patient to a high-volume center that elected to treat the patient with an endovascular stent-coiling procedure.

In summary, when treating patients with aSAH it is essential to act quickly and maintain cerebral perfusion pressure. In order to prevent rebleeding, the most rational approach to treatment of ruptured aneurysms combines surgical and endovascular treatments.[21–30,38] Patients with good neurologic grade should be treated as soon as possible with either technique to prevent re-rupture. Patients with Hunt and Hess classification scores of 4 and 5 at presentation may not do well, and the prognosis should be discussed with consultants and the patient and his/her family.

Endovascular coiling is favored in older patients who have well-defined aneurysms and in those with poorer grade at presentation as this technique will have less morbidity in patients with other underlying medical problems. Surgical clipping is favored in younger patients with easily accessible aneurysms located at vascular branch points.[21–30] In all instances, an experienced neurosurgeon should be guiding this decision and strong consideration should be given to referral to a high-volume aneurysm center if time and clinical condition permit.

## UNRUPTURED INTRACRANIAL ANEURYSMS

With all of the imaging studies performed today one could be faced with a new situation. A 25-year-old well-appearing female presented with a mild headache. She had just completed an outpatient CT scan advised by her primary physician and this had revealed a possible small aneurysm in the anterior circulation. The primary physician referred her to the ED for treatment. At the recommendation of the neurosurgical consultant an emergent lumbar puncture was performed. The cerebrospinal fluid was normal, indicating that if the lesion was indeed an aneurysm then it was unruptured. This valuable piece of information allowed for safe ED discharge and outpatient follow-up. The fact that this was a small unruptured aneurysm in the anterior circulation led the neurosurgeon to believe that this patient had a good prognosis and could easily be managed in the outpatient setting.[31–34]

It is important to understand some of the natural history of this disease to see how this decision is made in the clinical arena. Unruptured intracranial aneurysms occur at a frequency of up to 1% of the general population.[31,32] Historically, from observational series of patients with multiple aneurysms previously treated for SAH, the risk of rupture appears to be approximately 0.5–2.5% per year or (1/200–2.5/100 per year); this is a significant risk. Because rupture of an intracranial aneurysm leading to SAH is such a devastating disease, accounting for death and disability in nearly two-thirds of those affected, it is reasonable to assume that one of the ways to improve outcome is to treat aneurysms before they rupture. But the question is whether this is necessary in all cases.

To date, the largest series that explores the question is the International Study of Unruptured Intracranial Aneurysms (ISUIA).[33,34] ISUIA is a Mayo Clinic–coordinated study. Its initial phase was a retrospective epidemiological cohort study from 1991–1998. This was followed by a prospective cohort study in 2000 looking at neurosurgical and endovascular treatment options.

In the retrospective phase, to study the possible natural history of the disease, researchers recruited patients in a neurosurgical clinic and went through their charts to see how long these patients had had their aneurysms and how long they had gone without bleeding. The results, reported in the NEJM (Dec. 1998), showed that the risk of rupture for anterior circulation aneurysms of less than <10 mm was as low as 0.05% per year or (1 per 2,000 per year). This low figure may also reflect the fact that those patients who presented to the clinic were more likely to attend follow-up than their unfortunate counterparts who may have bled and not survived.

The prospective phase reported in the Lancet (in 2003) reported an overall rate of bleeding for all aneurysms of 3% over 4 years or less than 1% per year.

What is important is that the study found that aneurysms in the posterior circulation with a size > 7 mm are at the highest risk of rupture. While controversy exists regarding the ideal management, there are a few important pearls and guidelines: Patients with small, asymptomatic, unruptured anterior intracranial aneurysms and no SAH could be treated conservatively, for their risk of rupture may be as low as 1 in 2,000 per year. Acute symptoms include ischemia, headache, seizures, and cranial neuropathies.[30–34]

Chronic neurological symptoms include headache, visual deficits, weakness, and facial pain. Aneurysms that are posterior and have a size > 7 mm are at the greatest risk of rupture, which may be as high as 2.5 per 100 per year and, therefore, prompt treatment should be strongly considered. Endovascular and combined surgical techniques should be considered in all patients to give them the best chance of survival and the lowest morbidity.[30–34]

The management of these patients is evolving and consultation with the neurosurgeon and constant communication is essential. Treatment will be on a continuum. There may be times when smaller asymptomatic aneurysms that have not ruptured are carefully followed and observed, while posterior aneurysms and those with symptoms and larger size are taken up for treatment sooner. Controversy does exist and one should consider referral to a high-volume aneurysm center if time and clinical condition allow.

## CONCLUSION

In summary, this article has focused on the clinical approach to ischemic stroke, intracerebral intraparenchymal hemorrhagic stroke, aSAH, ruptured intracranial aneurysms, and unruptured intracranial aneurysms. Important details in the diagnosis and management of these conditions were presented in order to stimulate further discussion with colleagues in neurology and neurosurgery so as to develop rational treatment plans.
